# Leucine-rich alpha-2 glycoprotein expression in gingival tissues with chronic periodontitis

**DOI:** 10.6026/973206300210900

**Published:** 2025-05-31

**Authors:** Ramya Bharathi Badipati, Srikanth Chintalapani, Purumandla Usha, Pushpalatha Tummakomma, Aishwarya Arya, Navya Muttineni, Haripriya Rajaram

**Affiliations:** 1 Department of Periodontics, Mallareddy Institute of Dental Sciences, Mallards Vishwavidyapeeth (Deemed to be University), Hyderabad, Telangana, India

**Keywords:** Biomarker, enzyme-linked immunosorbent assay (ELISA) inflammation, leucine-rich alpha-2 glycoprotein

## Abstract

Alpha-2 glycoprotein rich in leucine is frequently used to assess inflammation. Therefore, it is of interest to assess the expression of Lucien-Rich Alpha-2 Glycoprotein in the gingival tissues of patients suffering from chronic periodontitis. Hence, 70 patients were divided into 2 groups each of thirty-five each. Participants in the case group had chronic periodontitis, while those in the control group had clinically healthy periodontal tissue. Gingival Index (GI), Plaque Index (PI), Probing Pocket Depth (PPD), Gingival Bleeding Index (GBI) and Clinical Attachment Level (CAL) were assessed for each group. Alpha-2 glycoprotein rich in leucine was assessed in both groups. Results show a strong correlation between chronic periodontitis and leucine-rich alpha-2 glycoprotein (LRG).

## Background:

Gram-negative bacterial infections, including those caused by Porphyromonasgingivalis and Bacteroidesforsythus, are the main cause of human periodontal disorders. The host immune system and these bacteria may interact intricately to cause inflammatory conditions that lead to the loss of the collagenous tissues supporting the teeth. Periodontal diseases involve several inflammatory mediators, including prostaglandins, matrix metalloproteinases (MMPs), interleukin (IL) 1, IL 6, IL 8, and tumour necrosis factor α (TNF α) [1]. Traditional diagnostic methods like clinical attachment level, probing depth, and several indices like gingival index, plaque index, bleeding on probing do not implicate the present disease status [2]. Various biomarkers are reported in saliva. Hence salivary biomarkers were advised to assess the periodontal disease status. Biomarkers whether sired by normal healthy subjects or by subjects deliberated by specific systemic diseases are symbolic molecules that could be harnessed to surveil health status [3]. Inflammatory mediators consisting of interleukin IL-8, IL-6, and tumor necrosis factor - α have been shown to exalt the degeneration of inflamed periodontal tissue. Hence inflamed gingival tissue elucidating these disease mediators signifies their role in deciphering the periodontal disease pathogenesis [1]. The 50 kDa glycoprotein known as leucine-rich alpha-2 glycoprotein (LRG) has a leucine-rich motif. Haematopoiesis produces leucine-rich α-2-glycoprotein-1 (LRG1) [4]. The acute-phase response mediators'up regulate the expression of leucine-rich alpha-2 glycoprotein (LRG), a secretary type I acute-phase protein. Alpha-2 glycoprotein, which is rich in leucine, is frequently used to assess inflammation. High amounts of the leucine-rich alpha-2 glycoprotein are produced during inflammation by the liver as well as by hematopoietic cells, including macrophages and neutrophils. Through its modulation of TGF-β signaling and promotion of cellular proliferation, differentiation and angiogenesis, leucine-rich alpha-2 glycoprotein has been implicated in the pathophysiology of inflammation. Leucine-rich alpha-2 glycoprotein is induced by inflammatory mediators such as IL-6, IL-22, and Lipopolysaccharides which are also the stimulating factors for the propagation of periodontal disease [5].

Leucine-rich alpha-2 glycoprotein as an inflammatory marker in the disease activity has been evaluated in many diseases including ulcerative colitis and rheumatoid arthritis [6]. The gold standard of immunoassays is the enzyme-linked immunosorbent assay (ELISA), which is a labelled immunoassay. This very sensitive immunological test can recognize and quantify proteins, glycoproteins, antigens, and antibodies. Leucine-rich alpha-2 glycoprotein was found in gingival tissues using ELISA [7]. In their investigation, Yucel *et al.* assessed the levels of the tumour necrosis factor TNF-, interleukin (I, L)-, 6 and LRG in the serum of patients with stage 3 periodontitis both before and after non-surgical periodontal therapy [8]. Therefore, it is of interest to assess the expression of Lucien-Rich Alpha-2 Glycoprotein in the gingival tissues of patients suffering from chronic periodontitis.

## Methodology:

The laboratory evaluation was finished at a different location, whereas the cross-sectional human investigation was conducted at the Department of Periodontics at the Mallareddy Institute of Dental Sciences in Hyderabad. The institutional ethical council granted ethical approval (Ref No: IEC/MRIMS/59/2019). All participants gave their informed consent. Total 70 participants were divided into 2 groups with 35 samples each as; Case Group: Subjects with chronic periodontitis and Control Group: Subjects with clinically healthy periodontium. Inclusion Criteria for control group were; patients aged between 18 and 48years, systemically healthy subjects, indicated for crown lengthening and orthodontic extractions, No sites with probing depth and CAL measurements >3mm, and less than 10% of sites exhibiting bleeding on probing Patients with chronic periodontitis were excluded if they had at least two interproximal sites with a clinical attachment level of ≥6 mm and one or more interproximal sites with a probing depth of ≥5 mm (not on the same tooth). Both sexes and ages ranging from 35 to 70 were involved. Acute oral infections, smoking, patients taking steroids or long-term non-steroidal anti-inflammatory drug use, pregnant and lactating women, patients who had taken antibiotics within the 12 weeks prior to the study, and subjects who had received periodontal therapy within the previous 6 months were all excluded. Gingival Index (GI-LOE & SILNESS), Gingival Bleeding Index (GBI-Ainamo J Bay), Plaque Index (PI) (Sillness J and Loe H in 1964), Probing Pocket Depth (PPD), and Clinical Attachment Level (CAL) were among the clinical indicators that were evaluated for both groups. In order to distinguish between healthy and ill people, the UNC-15 probe was utilized. Gingival tissues were taken from the site of significant periodontal inflammation and damage in the chronic periodontitis group Gingival tissues from periodontal healthy areas were taken from the control group and submitted to ELISA analysis The amount of human leucine-rich alpha-2 glycoprotein 1 (LRG1) in gingival tissues is measured using this ELISA kit. The obtained data was statistically evaluated using SPSS software version 23.0 using Mann-Whitney U test at P<0.005.

## Results:

Figure 1 - (see PDF) indicates that, inter group comparison of the mean of the Plaque Index in the Case Group was 1.70 ± 0.42 and in the Control Group, the mean values were 0.56 ± 0.30. These findings indicated a statistically considerable (p=<0.001) dissimilarity among the case group and control group. Figure 2 - (see PDF) indicated, Inter group comparisons of the mean of the Gingival Bleeding Index in the Case Group were 67.57 ± 13.05 and in the Control Group, the mean values were 5.91 ± 2.47. These results showed a statistically considerable (p=<0.001) difference between cases and the control groups. Figure 3 - (see PDF) indicates that, Inter group comparisons of the mean of Gingival Index in the Case Groupwere1.65± 0.46 and in the Control Group, the average values were 0.64± 0.33. These findings indicated a statistically considerable (p=<0.001) variation between cases and controls.

Figure 4 - (see PDF) indicated intergroup comparison of the mean of probing pocket depth in Case Group was 8.94 ±1.14 and in Control Group the mean values were 2.54±0.51. These findings indicated a statistically significant (p=<0.001) variation among cases and control groups. Figure 5 - (see PDF) indicates intergroup comparison of the mean of Clinical Attachment Level in Case Group was 11.20±1.49 and in the control group, the mean values were 2.54±0.51. These findings indicated a statistically considerable (p=<0.001) variation between cases and controls. Figure 6 - (see PDF) indicates, Comparison of mean of Lucien-Rich Alpha-2 Glycoprotein in both groups was analyzed, the mean of tissue LRG levels in Case Group were 19.07 ± 9.28 and in Control Group the mean values were 3.34 ± 1.80. These results showed a statistically significant (p=0.001) difference between case and control. Figure 7 - (see PDF) indicates, Comparison of percentage of male and female in both groups. Case Group constitutes of 15 males *i.e* about 42.9% and 20 female that is 57.1% whereas in the Control Group 16 male subjects are constituting about 45.7% and 19 female that is about 54.3%. Figure 8 - (see PDF) indicates comparison of mean of age groups in both the groups was analyzed. The mean values of Case were 46.37 ± 11.14 and the mean values of control were 28.09 ± 6.55.

## Discussion:

The assay known as immunoassay is the enzyme-linked immunosorbent assay (ELISA). Among other things, this very sensitive immunological test may recognize and quantify antigens, proteins, antibodies, glycoproteins, and hormones [9]. Chronic inflammation is an intricate biological process that develops in response to infection and/or other triggers and leads to tissue injury. Chronic inflammation triggers the release of cytokines and chemokine's, causing leukocytes to migrate to periodontal tissues, where they play a crucial role in pathogen eradication by releasing mediators in local inflammatory response. The methods for identifying and highlighting periodontal risk utilizing objective indicators like biomarkers are being influenced by developments in diagnostic investigations for oral and periodontal diseases. In the biological sciences, disease biomarkers have started to be important for drug discovery, monitoring therapeutic effects, and diagnosis. The objective of the biomarkers is to facilitate early detection of disease progression and contribute to more effective treatment [10].

Biomarkers in periodontitis can be collected via biopsy or GCF and are then measured by immunoassay. Periodontal diseases are regulated by cytokines, chemokine's, and other biologic mediators, demonstrating their utility as diagnostic and prognostic tools. The diagnosis of active phases of periodontal disease through identification of disease-promoting inflammatory biomarkers helps in predicting the patients who are vulnerable for periodontal breakdown and provide early treatment interventions which aid in the better clinical management of the disease [3]. Higher concentrations of serum-derived molecules, vascular-derived cellular components of inflammation, and locally generated molecules from the gingival tissues are all present in the inflammatory exudate that develops as gingival inflammation worsens [11]. A coordinated reaction to inflammation, infection, or tissue damage is known as the acute phase response. The activation of acute-phase proteins involved in homeostasis is a key characteristic of this response [8]. Proteins of the complement, coagulation, fibrinolytic system and inflammatory mediators etc., whose serum concentration changes by at least 25% in response to inflammation are referred to as acute-phase proteins [12]. The liver is the primary site of production for these acute-phase proteins in response to inflammation and infection. The acute-phase response is mediated by cytokines such as IL-1, IL-6, IL-8, and tumour necrosis factor-α25. The cell most frequently thought to be responsible for starting the acute phase response is a tissue macrophage [12]. Activated macrophages are the source of cytokines, which operate both locally, and remotely to boost local tissue production and trigger the systemic acute phase reactants [13]. Serum amyloid a protein (SAA) and C reactive protein (CRP) are two major acute phase proteins that are elevated at least 1000 times above normal values. Acute-phase protein concentrations, such as those of CRP and SAA, give diagnostic and prognostic information since they are correlated with the degree of tissue damage and the severity of the disease [14]. A recent study done on Inflammatory Bowel disease has deciphered the beneficial role of LRG compared to the other biomarkers in determining the disease activity thereby denoting LRG as a promising surrogate for CRP [15]. A recent study examined the correlation of LRG in periodontitis by analysing GCF and blood concentrations of LRG, IL-6, and TNF-α in individual with stage-3 periodontitis. They observed significant relationships between GCF and serum LRG with the clinical parameters: probing depth, CAL, and GI. The GCF and serum levels of LRG considerably elevated in patients with periodontitis and it is accompanied by notable decreases in LRG levels. These were detected both locally and systemically in parallel to the clinical periodontal healing provided by nonsurgical periodontal treatment. They advocated that LRG could be a valuable acute phase protein in inflammatory conditions as it is produced locally by inflammatory cells in periodontal tissues. Moreover, LRG could serve as a definite biomarker of inflammation sites because of its local production [16]. In the present study, Gingival tissues were chosen over the GCF to determine the expression of LRG because the gingival fluid does not exactly express the markers present in the tissues with variations depending on the marker even in the local inflammatory conditions. In this study, periodontal tissues were not obtained during periodontal surgery, because inflammation will not be pronounced in those sites when compared with untreated periodontitis.

Gingival tissue samples are immediately transferred to a container with Krebs Ringer Bicarbonate Buffer solution. These vials were stored at 4°C and transported within 48hrs and in the laboratory, they were further stored at -800C and further analyzed using a commercially available Human lrg elisa kit. In the present study, all clinical parameters namely the mean PI, GI, gingival bleeding index, ppd and cal were statistically higher in the periodontitis group than in the healthy group. The LRG levels in gingival tissues of periodontitis patients were spiked compared to the gingival tissues in healthy controls. These findings are in concurrence with the study of Yucel *et al.* [16]. Furthermore, the findings of the present study were in accordance with the studies of Serada, (2012) [6] observed increased expression of LRG in inflamed mucosa of ulcerative colitis patients confirming that inflamed tissue can be a source of production of LRG. The current study showed an elevated expression of LRG in the deeper sites compared to the shallow sites. These results are in compliance with the study of Yucel *et al.* [16] where they have detected higher levels of IL-6 in deeper sites compared to the shallow sites which are because of the increased epithelial permeability due to progressive inflammation. In the present study increased expression of LRG in the deeper sites can be corroborated with the same. Ouhara *et al.* examined the synthesis of leucine-rich α-2-glycoprotein-1 (LRG1) in patients with periodontitis and its potential as a novel diagnostic marker for the condition. They determined that LRG1 levels in serum and periodontal tissue are elevated in periodontitis and contribute to periodontal tissue damage via interleukin-6 production [4]. Igarashi *et al.* proposed a correlation between salivary LRG levels and the severity of periodontitis. LRG was recognised as a biomarker for assessing disease activity in autoimmune disorders [17]. Yucel *et al.* examined the levels of LRG, interleukin (IL)-6, and tumour necrosis factor (TNF)-α in gingival crevicular fluid (GCF) and serum of patients with Stage 3 periodontitis, both prior to and following non-surgical periodontal treatment. The researchers determined that LRG expression was elevated in Stage 3 periodontitis, both locally and systemically, and that non-surgical periodontal therapy effectively reduced LRG levels [16]. Serada and colleagues Serum LRG levels were quantified using enzyme-linked immunosorbent assay (ELISA) in patients with ulcerative colitis (UC) and healthy controls (HC) and assessed for connection with disease activity. They concluded that serum LRG concentrations were considerably increased in active UC patients compared to those in remission [6]. Sumedha *et al.* colleagues employed immunohistochemistry to measure and compare the expression of Toll-like receptor 2 (TLR2) and cluster of differentiation 14 (CD14) in gingival tissues, revealing a strong association between TLR2 and CD14 expression levels and the severity grades of chronic periodontitis [18]. To the best of our knowledge and available evidence, there is no clinical data on the expression of Lucien-rich alpha-2 glycoprotein in the gingival tissues. This is the first study to evaluate the production of LRG locally in periodontal tissues of chronic periodontitis. The results of the current research demonstrated that LRG is a useful biomarker of periodontal disease activity. Within the limitations of the present study, it has been revealed that LRG expression is more in gingival tissues of chronic periodontitis patients compared to the healthy subjects, thereby suggesting its role in the pathogenesis of chronic periodontitis. A major limitation in the present study is that the patients were categorized into groups based on the clinical parameters and radiographs only and not on microbiologic and histologic analysis. Furthermore, the expression of LRG in different stages of periodontitis has not been delineated. Additional researches with a large sample size to validate our results.

## Conclusion:

There was an increased expression of LRG in gingival tissues of chronic periodontitis patients while decreased expression of LRG in gingival tissues of healthy subjects. Thus, a significant relationship between leucine-rich alpha-2 glycoprotein and chronic periodontitis is shown. Hence, LRG is a useful biomarker for assessing the disease severity.

## References

[1] Noh M.K (2013). Experimental and Therapeutic Medicine..

[2] Ramenzoni L.L (2021). Diagnostics (Basel)..

[3] Giannobile W.V (2009). Periodontol 2000..

[4] Ouhara K (2024). Oral Diseases..

[5] Naka T (2018). Immunol Med..

[6] Serada S (2012). Inflamm Bowel Dis..

[7] Sakamoto S (2018). J Nat Med..

[8] Keles Yucel Z.P (2021). J Periodontol..

[9] Hnasko R (2015). Methods in molecular biology..

[10] Graves D.T (1999). Clin Infect Dis..

[11] Liu C.H (2017). Nat Immunol..

[12] Polepalle T (2015). J ClinDiagn Res..

[13] Duque G.A (2014). Front Immunol..

[14] Schultz D.R (1990). Semin Arthritis Rheum..

[15] Naka T (2018). Immunol Med..

[16] Yucel Z.P.K, Balli U. (2021). J Periodontol..

[17] Igarashi N (2025). Journal of Oral Science..

[18] Sumedha S (2017). Biotech Histochem..

